# Vertebral Osteomyelitis: An Under-Recognized Infectious Complication in Patients on Home Parenteral Nutrition

**DOI:** 10.14740/jocmr1825w

**Published:** 2014-05-22

**Authors:** Genevieve Huard, Mickael Bouin, Michel Lemoyne, Louise D’Aoust

**Affiliations:** a Gastroenterology and Nutrition Unit, Hopital Saint-Luc, Centre Hospitalier de l’Universite de Montreal, Montreal, Canada

**Keywords:** Vertebral osteomyelitis, Parenteral nutrition, Infectious complications

## Abstract

**Background:**

Patients on home parenteral nutrition (HPN) are at high risk of central venous catheter sepsis (CVCS). CVCS can be associated with distant bacterial seeding. However, few cases of vertebral osteomyelitis (VO) related to HPN have been reported. For this reason, we made the hypothesis that the incidence of VO in patients on HPN is probably higher than what is reported. The goal of this study was to evaluate the incidence of infectious complications, and more specifically, the incidence of VO in patients on HPN.

**Methods:**

A retrospective study of all patients receiving HPN from 2001 to 2006 was conducted. Patients who received HPN for < 1 month were excluded. Infectious complications and, more specifically, cases of VO were searched.

**Results:**

Thirty-one patients received HPN and were included in the analysis. Forty-four infectious complications occurred (1.302/1,000 CVC-days). The most frequent infectious complication was urinary tract infection (25 cases; 0.740/1,000 CVC-days). Seven CVCS occurred in five different patients (0.207/1,000 CVC-days). In patients with CVCS, 42.9% (three cases) developed a secondary VO. No predictive factors for the development of VO could be identified in univariate analysis.

**Conclusion:**

We report a very low rate of infectious complications and an even lower rate of CVCS in patients on HPN. However, we report that 42.9% of our cases of CVCS developed a secondary VO. Consequently, VO must be part of the differential diagnosis among patients with HPN who complain of back pain.

## Introduction

Patients with short bowel syndrome (SBS) needing long-term nutritional support with home parenteral nutrition (HPN) are at high risk for infectious complications [1]. The presence of an intra-vascular device, a low-grade systemic inflammation, an altered mucosal immune response, a diminished intestinal barrier function and a possible systemic immunocompromise is thought to contribute to the infectious susceptibility encountered in this group of patients [1]. It is also recognized that infectious complications in patients on HPN are associated with high morbidity and mortality rates and with high hospital costs [2, 3].

Central venous catheter-related sepsis (CVCS) is the most frequent infectious complication encountered in patients on HPN [1, 2, 4, 5]. The incidence rate of CVCS in patients on HPN is estimated to be between 3 and 6/1,000 days of central venous catheterization (CVC) [2, 4-7]. The recognized risk factors for the development of a catheter-related sepsis are: a long duration of HPN, the type of disease leading to SBS and the presence of a port-A-Cath [4]. CVCS often needs to be treated with antibiotic therapy, they can lead to the removal of the central catheter device and they can cause venous thrombosis and subsequent vascular access lost [2, 3]. They are also associated with long hospital stay and high hospital costs [2, 3]. Furthermore, CVCS can be associated with distant bacterial seeding causing infection of distant organs or tissues. Even if CVCS can be associated with hematogenous spread, only few cases of vertebral osteomyelitis (VO) related to HPN are reported in the literature [2, 8].

VO is a rare disease with a reported incidence of 1/250,000 to 1/450,000 [9, 10]. Hematogenous spread of a bloodstream infection is, by far, the most common mechanism leading to the development of VO [10]. The diagnosis of this rare infection is often delayed and could be underestimated because signs and symptoms are usually insidious [9, 11]. Since patients on HPN are at high risk for osteoporotic fractures, back pain is a frequent complain in this group of patients and the diagnosis of VO can easily be overlooked [12]. For this reason, we made the hypothesis that the incidence of VO in patients on HPN is probably under-reported in the current literature. The goal of this study was to evaluate the incidence of infectious complications, and more specifically, the incidence of VO in patients on long-term HPN followed at our single HPN clinic.

## Methods

### Patients

We reviewed the medical records of consecutive patients aged 18 years and older referred from January 1, 2001 to December 31, 2006 to our single HPN clinic (Hopital Saint-Luc, Universite de Montreal, tertiary medical center with expertise in nutritional support). Our institutional board review approved the study. Patients followed at the HPN clinic were included if they received HPN for at least 1 month during the study period. All patients included received HPN for intestinal failure, received standardized training sessions including information about infection prevention and were followed at least once a month by a specialized team in nutritional support. Patients who received HPN for less than 1 month and patients receiving only hydroelectrolytic perfusions were excluded.

### Infections

Infectious complications were searched in the medical records of each patient. The diagnosis of urinary tract infection was established when the following criteria were present: new onset of urinary symptoms (dysuria, frequency, urgency, suprapubic pain or hematuria), presence of leucocytes on the urinalysis and a positive urine culture [13-15]. The diagnosis of pneumonia was made when clinical signs and symptoms compatible with this diagnosis (cough, fever, dyspnea, sputum production, and so on) were present and a pulmonary infiltrate was demonstrated on the chest X-ray [16, 17]. The diagnosis of a CVCS was established when signs and symptoms of sepsis (fever, chills and/or hypotension) were present in a patient with a central venous catheter (if it has been removed, the signs and symptoms must have begun during the first 48 h following the catheter removal) [18-21]. The diagnosis of CVCS also needed positive blood cultures for the same microorganism obtained from a peripheral venipuncture and from the central venous catheter [20, 21]. Finally, the diagnosis of VO related to a CVCS was established when CVCS criteria were met in a patient with vertebral pain associated with fever (> 38 ^°^C) and a confirming imaging study (computed tomography (CT) scan, magnetic resonance imaging (MRI) and gallium scanning) [22-24].

### Data analysis

All quantitative data were expressed as a mean ± standard deviation (SD). Univariate analyses were conducted to determine if risk factors could predict an increased risk of VO. P value < 0.05 were considered significant.

## Results

A total of 31 patients were followed at our single HPN clinic between January 1, 2001 and December 31, 2006. All patients met the inclusion criteria and no patient was excluded.

### Patients

Twenty-tree patients (74.2%) were females and the mean age of the population was 52.3 ± 10.4 years. The mean weight of the cohort was 56.1 ± 10.2 kg and the mean BMI was 21.4 ± 2.9 kg/m^2^. The main reason for initiation of HPN in our population was SBS related to multiple surgeries for Crohn’s disease in nine patients. Other reasons for initiation of HPN were surgical SBS (seven abdominal cancers, four intestinal volvulus, three intestinal ischemia and one Gardner syndrome) and functional SBS (three scleroderma, two auto-immune enteritis and two radiation enteritis). During the study period, we had a total of 33,772 days of HPN and the mean number of days of HPN was 1,125.7 ± 694.7 days.

### Infections

We identified 44 infectious complications (1.302/1,000 CVC-days) (Table 1).

**Table 1:** Infectious Complications in Patients on HPN

Type of infection	Nb infection (nb patients)	Nb infection per 1,000 CVC-day	% Total complication
Urinary tract infection	25 (10)	0.740	56.8
Pneumonia	8 (6)	0.237	18.2
CVCS	7 (5)	0.207	15.9
Secondary vertebral osteomyelitis	3 (3)	0.089	
Sinusitis	1 (1)	0.029	2.3
Dental abscess	1 (1)	0.029	2.3
C. Difficile colitis	1 (1)	0.029	2.3
Bacterial translocation with septicemia	1 (1)	0.029	2.3
Total	44	1.302	100

Twenty-five urinary tract infections were identified in ten patients. Thirteen of those urinary tract infections occurred in the same patient, who had a past medical history of retroperitoneal fibrosis with secondary hydronephrosis. Eight pneumonia were diagnosed in a total of six different patients. Furthermore, seven primary catheter infections (0.207/1,000 IVD-days) were diagnosed in five different patients. The isolated microorganisms during those CVCS were Staphylococcus epidermidis (n = 3; 42.9%), Candida sp. (n = 2; 28.6%), Staphylococcus lugdunensis (n = 1; 14.3%) and polymicrobial (n = 1; 14.3%). Three patients (42.9%) with a CVCS developed a VO. We also identified one case of each of the following infections: sinusitis, dental abscess, clostridium difficile colitis and septicemia due to bacterial translocation. All three cases of VO were related to a primary catheter infection as demonstrated by the diagnosis of a concomitant CVCS. Table 2 shows the characteristics of those individuals.

**Table 2:** Characteristics of Individuals With VO

	Patient 1	Patient 2	Patient 3
Age (years)	42	58	79
Main disease	Crohn with short bowel syndrome (under 6-MP)	Auto-immune enteropathy with malabsorption (under infliximab)	Radiation enteritis
Duration of HPN (months)	12	1	19
Catheter characteristics			
Type	Port-A-Cath	Port-A-Cath	Hickmann
Site	Right internal jugular vein	Right subclavian vein	Right internal jugular vein
Duration (months)	13	1	19
Clinical criteria			
Back pain	Yes (severe pain)	Yes (slight)	Yes (slight)
Temperature > 38 °C	Yes	Yes	Yes
Duration of symptoms	1 month before consultation	1 week before presentation	2 weeks before presentation
Spine imaging studies			
Computed tomography	Not performed	Possible Spondylodiscitis at L4-L5	Not performed
Magnetic resonance imaging	Spondylodiscitis at D7-D8 and D9-D10	Possible Spondylodiscitis at L4-L5	Sondylodiscitis at L3-L4
Additional imaging			
X-ray	Osteopenia, compression fractures of D7 and D8	Osteopenia, no spondylodiscitis	Osteopenia, no spondylodiscitis
Gallium scanning	Spondylodiscitis at D9-D10	Spondylodiscitis at L4-L5	Spondylodiscitis at L4
Cardiac ultrasound	No endocarditis	Not performed	No endocarditis
Microbiological criteria			
Peripheral vein cultures	Staphylococcus lugdunensis	Staphylococcus epidermidis	Staphylococcus epidermidis
Central catheter cultures	Same as peripheral vein	Not performed	Same as peripheral vein cultures
Treatment			
IV antibiotics for 6 weeks	Cefazolin	Vancomycine	Vancomycine
Central catheter replacement	Yes	Yes	Yes

In univariate analysis, no predictive factor for the development of VO was identified in patients with osteomyelitis in comparison with those without osteomyelitis: mean age (61.8 ± 18.4 vs. 51.1 ± 12.5 years; P = 0.5), mean BMI (25.6 ± 3.4 vs. 20.9 ± 3.7 kg/m^2^; P > 0.5) and mean duration of HPN (1,054 ± 716 vs. 1,133 ± 806.6 days; P > 0.5). No predictive factor was identified for the development of CVCS when patients with CVCS were compared with patients without CVCS: mean age (56.73 ± 13.7 vs. 50.5 ± 13.1 years; P = 0.26), mean BMI (28.6 ± 13.2 vs. 23.1 ± 8.4 kg/m^2^; P > 0.5) and mean duration of HPN (1,011 ± 683 vs. 1,167 ± 832 days; P > 0.5). No difference was seen between patients with simple CVCS and those who developed a VO due to a CVCS: mean age (53.1 ± 10.1 vs. 56.0 ± 15.2 years; P > 0.5), mean BMI (21.2 ± 1.9 vs. 25.6 ± 3.4 kg/m^2^; P = 0.07) and mean duration of HPN (986 ± 747 vs. 1,054 ± 716 days; P > 0.5).

## Discussion

In this retrospective study, we report a total of 44 infectious complications that occurred during a period of 33,772 days of HPN. This number of complications represents an infection rate of 1.302/1,000 CVC-days. More than half (56.8%) of those infectious complications were in fact urinary tract infections, which were probably not related to HPN itself. Furthermore, 52% of those urinary tract infections occurred in the same patient who had obstructive uropathy related to retroperitoneal fibrosis.

Our rate of CVCS (0.207/1,000 CVC-days) is very low compared to the reported rates of CVCS in the HPN literature (3-6/1,000 CVC-days) [2, 4-7]. We explain this result by the fact that all patients received personalized learning sessions, by a nurse practioner, on infection prevention during the training for HPN. Also all patients were closely followed in a single tertiary center with an expertise on nutritional support. Finally, the low incidence of CVCS could also be, in part, explained by the fact that only patients who had positive blood cultures obtained from the central catheter and from a peripheral venipuncture were considered as having a primary catheter infection to explain the bacteremia, as suggested in the most recent literature. To be strictly adherent to the suggested diagnostic criteria of CVCS, seven cases of positive blood cultures obtained from a peripheral venipuncture that proved to have sterile blood cultures obtained from the central catheter were not considered as CVCS. Even if we include those seven cases of bacteremia that does not fulfill the diagnostic criteria for CVCS, we would have obtained a rate of 0.415/1,000 CVC-days, which is still lower than the reported rates in the HPN literature.

In our study, we also report three cases of VO related to hematogenous spread from infected catheters. In fact, 42.9% of patients with a confirmed CVCS developed a secondary VO after bacterial seeding in the spine. This represents a rate of 0.089 VO/1,000 CVC-days, which is relatively high, considering that only few cases of hematogenous osteomyelitis are reported in the HPN literature [2, 8]. No predictive factor for the development of VO could be identified in univariate analysis and this is probably related to the small number of patients included in this study. However, we postulate that these patients were predisposed to develop VO under HPN because of the use of immunosuppressive treatment (patients 1 and 2), multiple catheter manipulations by different caregivers (patient 2), long delay (> 1 week) between onset of symptoms and medical consultation (patients 1 and 3), osteopenia (patients 2 and 3) and osteoporosis with compression fractures (patient 1). All three cases of VO were attributed to a primary catheter infection because the same strain of bacteria was isolated from the central catheter and the peripheral blood cultures. Also, the isolated bacteria were known to be skin germs and possible cause of CVCS. However, in patient 2, central venous blood cultures were not performed. We postulated that the bacteremia in this patient was related to a catheter infection since the patient had no other possible site of infection to explain this persistent bacteremia and because multiple peripheral blood cultures were positive for the same bacteria (n = 5).

The possible limitations of this study are the small sample size (n = 31), its retrospective nature and the absence of central catheter blood cultures in patient 2 which would have proved the hematogenous spread from an infected catheter. However, our study is the biggest published to date on VO related to HPN. Our patients were systematically followed at an HPN clinic in a tertiary center and all patients presenting with signs of infection and vertebral pain had spine imaging studies to exclude a VO.

### Conclusions

To our knowledge, we report a very low rate of infectious complications in our HPN patients. Furthermore, we report a much lower rate of CVCS compared to the published literature on HPN. We also report that almost 43% of all CVCS developed a secondary VO. This is the first report in the literature of such a high rate of VO in patients on HPN with CVCS. Indeed, it is important to remember that VO can be an infectious complication of HPN. This diagnosis should always be suspected when a patient on HPN complains of back pain and fever. Any suspicion of this diagnosis justifies conducting peripheral and catheter blood cultures as well as a spine imaging study.

## Clinical Relevancy Statement

Patients with SBS on HPN are at high risk for infectious complications. The finding that, in this study, 42.9% of the CVCS were complicated by a secondary VO is very important. This complication must be incorporated to the differential diagnosis of the physicians evaluating patients on HPN complaining of fever or back pain.
